# Supplementary material for: The GOLIATH Project: Towards an Internationally Harmonised Approach for Testing Metabolism Disrupting Compounds
Source: Int J Mol Sci. 2020 May 14;21(10):3480. doi: 10.3390/ijms21103480 (PMC7279023; doi:10.3390/ijms21103480)
Supplement: Supplementary file 1 [file ijms-21-03480-s001.pdf]

International Journal of
Molecular Sciences

MDPI

Project report

The GOLIATH project: Towards Internationally Harmonised Approaches for Testing Metabolism Disrupting Compounds

SUPPLEMENTARY TABLES AND FIGURES

International Journal of *Molecular Sciences*

MDPI

Supplementary Table 1: Overview of the reported associations between (early life) exposure to the initial set of GOLIATH chemicals and metabolic disorders in human epidemiological studies. For PFOA, TCS, p,p'-DDE, and BPA, studies of 2016 onwards were selected.

Chemical	Endpoint	Epidemiologic design	Population	Statistically significant associations / Findings	First Author + Year of publication
**TBT**	**Growth and Ponderal Index**	Prospective (Outcome ~ TBT placenta)	Newborns followed in childhood, Finland	Weight gain during the first three months of life (positive); no significant associations between placenta OTC concentrations and child length, weight or PI at any time point were found	Rantakokko, 2014 [1]
**PFOA**	**Liver disease**	Cross-sectional	Adults, China	Serum adipocytokines: TNF-alpha (negative); CK18 M30 (positive)	Bassler, 2019 [2]
**PFOA**	**Gestational diabetes (GD)**	Prospective: GD ~ PFOA in early pregnancy	Healthy US women	Positive association with GDM among women with a family history of T2D	Rahman, 2019 [3]
**PFOA**	**Overweight**	Cross-sectional	Adults, China	Higher prevalence of overweight and positive association with waist circumference, more pronounced in women	Tian, 2019 [4]

Chemical	Endpoint	Epidemiologic design	Population	Statistically significant associations / Findings	First Author + Year of publication
**PFOA**	**Lipids/lipoproteins**	Cross-sectional	NHANES 2005-2014, adults	Total (positive) and LDL cholerterol (positive). Greater susceptibility to elevated total and LDL cholesterol in obese participants, with differences between men and women.	Jain, 2019 [5]
**PFOA**	**Childhood adiposity**	Meta-analysis of Prospective cohorts	10 cohort studies, N = 6076	Childhood body mass index (positive)	Liu, 2019 [6]
**PFOA**	**Birth antropometric measures**	Cross-sectional	Newborns, US (KIDS)	No significant associations	Bell, 2019 [7]
**PFOA**	**Type 2 diabetes**	Prospective cohort: T2D ~ dietary exposure	Adults, France (E3N)	U-shape association	Mancini, 2019 [8]
**PFOA**	**Glucose related outcomes**	Prospective birth cohort: outcome gestational week 28 ~ PFOA gestational week 11	Pregnant women, Denmark (Odense Child Cohort)	PFOA was not associated with glucose related outcomes (other PFASs were)	Jensen, 2018 [9]
**PFOA**	**Cord blood DNA methylation**	Cross-sectional	Mothers and newborns, Japan (Sappora cohort, Hokkaido Study)	DMP: ZBTB7A, USP2-AS1, TCP11L2, NTN1; DMR: ZFP57, CYP2E1, SMAD3, SLC17A9, GFPT2, DUSP22, and TCERG1L	Miura, 2019 [10]
**PFOA**	**Type 2 diabetes**	Prospective nested case-control	Adults, US, NHSII	Risk of type 2 diabetes (positive)	Sun, 2018 [11]

Chemical	Endpoint	Epidemiologic design	Population	Statistically significant associations / Findings	First Author + Year of publication
**PFOA**	**Body weight and resting metabolic rate**	Randomnised clinical trial	Adults, US	Participants lost an average of 6.4 kg of body weight during the first 6 months (weight-loss period) and subsequently regained an average of 2.7 kg of body weight during the period of 6-24 months (weight regain period).Examine associations of PFAS exposure with changes in body weight and resting metabolic rate (RMR) in a diet-induced weight-loss setting. Higher baseline levels of PFASs were significantly associated with a greater weight regain, primarily in women	Liu, 2018 [12]
**PFOA**	**Lipids (total cholesterol, HDL, LDL) and alanine aminotransferase**	Prospective cohort: outcome childhood ~ prenatal exposure maternal plasma preganancy	Children, US	Higher TC and LDL in girls; Higher ALT in boys and girls; Lower ALT in boys and girls (the latter two predict better cardiovascular health)	Mora, 2018 [13]
**PFOA**	**Metabolic outocmes: impaired glucose tolerance (IGT), gestational diabetes mellitus (GDM),**	Prospective/cross-sectional: outcome ~PFOA 1st trimester	Pregnant women, Spain (INMA)	total cholesterol (positive)	Matilla-Santander, 2018 [14]

Chemical	Endpoint	Epidemiologic design	Population	Statistically significant associations / Findings	First Author + Year of publication
	**first-trimester serum levels of triglycerides, total cholesterol, and C-reactive protein (CRP)**				
**PFOA**	**Metabolic Syndrome**	Cross-sectional	Adults, China	increased risk of metabolic syndrome, systolic blood pressure (positive), hypertriglyceridemia (positive), obesitry (positive)	Yang, 2018 [15]
**PFOA**	**Bone density; cardio-metabolic risk factors**	Cross-sectional	Children, 2017	Total and LDL cholesterol (positive)	Khalil, 2017 [16]
**PFOA**	**Glycemic indicators and diabetes index**	Prospective	Diabetes Prevention Program Trial, US	Evaluated adjusted associations for plasma PFAS concentrations with diabetes incidence and key glycemic indicators measured at baseline and annually over up to 4.6 y. Baseline: homeostatic model assessment of insulin resistance (HOMA-IR) (positive); β-cell function (HOMA-β) (positive); fasting proinsulin (positive); glycated hemoglobin (HbA1c) (prositive)	Cardenas, 2017 [17]

Chemical	Endpoint	Epidemiologic design	Population	Statistically significant associations / Findings	First Author + Year of publication
**PFOA**	**Weight gain; BMI; Waist circumference; blood pressure**	Prospective	Childran, Spain (INMA)	Little or no evidence of associations between low prenatal PFAS exposures and outcomes related to cardiometabolic risk in a cohort of Spanish children followed from birth until 7 y	Manzano-Salgado, 2017 [18]
**PFOA**	**Biochemistry profiles of metabolic syndrome**	Cross-sectional	Adults, US (NHANES 2013-2014)	Increased linear PFOA was associated with increases in total cholesterol, serum albumin and an enhancement of beta cell function as well as a decrease in the serum globulin. Increased branched PFOA was significantly associated with increased fasting glucose. All isomers of PFOA were positively associated with high-density lipoprotein-cholesterol (HDL-C) and negatively associated with glycohemoglobin (HbA1C).	Liu, 2018 [19]
**PFOA**	**Birth antropometric outcomes and maternal glucose and lipids**	Prospective: Outcome ~ PFOA mid pregnancy	Mothers and newborns, US (Healthy Start)	Birth weight (negative); Adipositiy (negative); maternal glucose mid pregnancy (negative). Mediation effect of maternal glucose on	Starling, 2017 [20]

Chemical	Endpoint	Epidemiologic design	Population	Statistically significant associations / Findings	First Author + Year of publication
				association between PFAS and neonatal adiposity	
**PFOA**	**DNA methylation cord blood**	Prospective: Outcome ~ PFOA mid pregnancy	Mothers and newborns, US	among top20 genes: RASA3; OPRD1	Kingsley, 2017 [21]
**PFOA**	**Body fat at age 9 in girls**	Prospective: Outcome ~ PFOA maternal mid pregnancy	Mothers and children, UK (Avon)	BF age 9 (positive)	Hartman, 2017 [22]
**PFOA**	**Fetal growth**	Prospective case-cohort: outcome ~PFOA early second trimester	Mothers and newborns, Sweden and Norway	Higher odds for small for gestational age (SGA) birth	Lauritzen, 2017 [23]
**PFOA**	**Cord blood transcriptome**	Cross-sectional	Newborns, Belgium	Transcription factor enrichment analysis: Progesteron Receptor Signaling; Gene: ICA1; Cell signaling: Natural killar cell signaling	Remy, 2016 [24]
**PFOA**	**Metabolic function**	Prospective (Outcome ~ PFOA early pregnancy)	Mothers and children, US (Project Viva)	HOMA-IR (negative), more pronounced in females	Fleish, 2017 [25]
**PFOA**	**Adiposity and glucose metabolism**	Prospective	Children followed until young adulthood, Denmark (European Youth Heart Study)	PFOA exposure in childhood was associated with decreased β-cell function at 15 years of age. No associations observed between exposure during adolescence and	Domazet, 2016 [26]

Chemical	Endpoint	Epidemiologic design	Population	Statistically significant associations / Findings	First Author + Year of publication
				indicators of adiposity and glucose metabolism in young adulthood.	
**PFOA**	**Adiposity**	Prospective (Outcome ~ PFOA early pregnancy)	Mothers and children, US (Project Viva)	In girls, mid childhood (7 tears): Body mass index (positive), subscapular and triceps skinfold thickness (positive), DXA total fat mass index (positive); no associations for boys and early childhood	Mora, 2017 [27]
**PFOA**	**Beta cell deficient diabetes**	Cross-sectional	Adults, US (C8 health project)	Diabetes (negative), strongest for type 1 diabetes	Conway, 2016 [28]
**PFOA**	**Glucose tolerance and diabetes**	Cross-sectional	Adults, Taiwan	Potential protective effect against glucose intolerance and the risk of diabetes	Su, 2016 [29]
**PFOA**	**Adiposity**	Prospective (Outcome ~ PFOA pregnancy)	Mothers and children, US (HOME study)	Adiposity 8y (positive), waist circumference 8y (positive), BMI gain 2y-8y (positive)	Braun, 2016 [30]

**Chemical**	**Endpoint**	**Epidemiologic design**	**Population**	**Statistically significant associations / Findings**	**First Author + Year of publication**
**TCS**	**Blood pressure (BP)**	Prospective birth cohort (Outcome and TCS measured in 3 trimesters of pregnancy)	Pregnant women, China	In the women carrying male fetuses, urinary TCS concentrations were associated with a slight change of SBP during pregnancy. In the women carrying female fetuses, no chemical was associated with SBP, while urinary concentration of triclosan was inversely associated with DBP, though the magnitude was small.	Liu, 2019 [31]
**TCS**	**Fetal and early childhood growth**	Prospective birth cohort (Outcome ~ TCS 3 trimesters of pregnancy	Mothers and children, China	Girls: third-trimester estimated fetal weight (increase), 2 year-old weight z-score (increase); early and middle stage of pregnancy may be the windows of vulnerability	Wu, 2019 [32]
**TCS**	**Child adiposity**	Prospective birt cohort (Outcome at 8 y ~ TCS during pregancy and annually from 1-5 years and at 8 years)	Mothers and children, US	Girls: Child adiposity at 8y ~ Prenatal triclosan	Kalloo, 2018 [33]
**TCS**	**Gestational diabetes (GD) and birth weight**	Cross-sectional	Pregnant women, China	Birth weight (positive, female infants)	Ouyang, 2018 [34]
**TCS**	**Fetal growth**	Prospective birth cohort (Outcome ~ TCS during	Mothers and newborns, US (LIFECODES)	Estimated fetal weight and birth weight (negative, male infants)	Ferguson, 2018 [35]

Chemical	Endpoint	Epidemiologic design	Population	Statistically significant associations / Findings	First Author + Year of publication
		pregnancy)			
**TCS**	**Gestational diabetes, impaired glucose tolerance, gestational weight gain, fetal markers of metabolic function**	Prospective birth cohort (Outcome ~ TCS first trimester pregnancy)	Mothers and newborns, Canada (MIREC)	No support of an association between triclosan concentrations in pregnancy and fetal metabolic markers, glucose disorders of pregnancy, or excessive gestational weight gain	Shapiro, 2018 [36]
**TCS**	**Birth outcomes**	Cross-sectional	Mothers and newborns, China	No statistically significant associations after adjustment for covariates	Huo, 2018 [37]
**TCS**	**Adiposity**	Prospective (Outcome up to 15 years ~ TCS 6-8y)	Children followed until 15 y, US	Adiposity (posity, only among overweight girls)	Deierlein, 2017 [38]
**TCS**	**Birth outcomes**	Prospective (Outcome ~ TCS 3rd trimester pregnancy)	Mothers and newborns, US	No associations oberserved for TCS	Geer, 2017 [39]
**TCS**	**Childhood fat mass**	Prospective (Outcome ~ TCS 3rd trimester pregnancy)	Mothers and children, US	No assosiation observed for TCS	Buckley, 2016 [40]
**TCS**	**Birth athropometric measures**	Prospective (Outcome ~ TCS pregnancy)	Mothers and newborns, Denmark (Odense Child Cohort)	Head circumference (negative, boys); abdominal circumference (negative, borderline signifcant boys)	Lassen, 2017 [41]

Chemical	Endpoint	Epidemiologic design	Population	Statistically significant associations / Findings	First Author + Year of publication
**p,p'-DDE**	**Blood pressure; hypertension**	Repeated measures of Outcome and Exposure 10 years apart	Adults, Sweden (Västerbotten Intervention Programme)	DDE levels were significantly associated with odds of hypertension when BMI was not included in the model as a covariate.	Donat-Vargas, 2018 [42]
**p,p'-DDE**	**Type 2 Diabetes**	Prospective (Outcome ~p,p'-DDE 11 years earlier)	Adults, US (Nurses' Health Study II)	T2D (positive, upper vs lower tertile exposure)	Zong, 2018 [43]
**p,p'-DDE**	**Body weight; body composition**	Prospective (Outcome 1-2 y ~ p,p'-DDE mother near delivery)	Children, South Africa (VHEMBE)	p,p'-DDT, girls: body composition (positive); body weight (positive)	Coker, 2018 [44]
**p,p'-DDE**	**Obesity**	Prospective (Outcome 70-75-80y ~ Exposure 70y)	Adulsts, Sweden (PIVUS)	Fasting glucose (postive), BMI (positive), hypertension (positive)	La Merrill, 2018 [45]
**p,p'-DDE**	**Obesity**	Systematic Review; Meta-analysis	7 Prospective Studies for meta-analysis	BMI z-score (positive)	Cano-Sancho, 2017 [46]
**p,p'-DDE**	**Adiposity**	Prospective (Outcome 12y ~ p,p'-DDE prenatal mother)	Children, US (CHAMACOS)	12y, boys: o,p'-DDT, p,p'-DDT, and p,p'-DDE: BMI z-score (positive)	Warner, 2017 [47]
**p,p'-DDE**	**Metabolic Syndrome**	Cross-sectional	Adults, US (Anniston Community Health Survey)	Metabolic Syndrom (positive, for p,p'-DDT across multiple quintiles; for p,p'-DDE for the highest quintile relative to the first)	Rosenbaum, 2017 [48]
**p,p'-DDE**	**Gestational diabetes, birth size**		Pregnant women, Faroe Islands	Gestational diabetes (positive); head circumference (positive)	Valvi, 2017 [49]

Chemical	Endpoint	Epidemiologic design	Population	Statistically significant associations / Findings	First Author + Year of publication
**p,p'-DDE**	**Diabetes**	Cross-sectional	Adults, Canadian Arctic, Adult Inuit Health Survey	Diabetes (positive, highest versus mowest quartile); Fasting glucose (positive, highest versus mowest quartile)	Singh, 2017 [50]
**p,p'-DDE**	**Disruption adipose tissue oxidative microenvironment**	Cross-sectional	> 16 years, Spain	lipid peroxidation (TBARS, positive); SOD acitivity (positive)	Artacho-Cordón, 2016 [51]
**p,p'-DDE**	**Birth Outcomes**	Cross-sectional	Newborns, China	birth weigth (positive)	Xu, 2017 [52]
**p,p'-DDE**	**Metabolomics**	Intervention study	Adults, UK (FoodCAP research project)	Sphingolipids and Glycerophospholipids lipids families were identified and found significantly ($p < 0.05$) different between high and low POPs exposure levels.	Carrizo, 2017 [53]
**p,p'-DDE**	**Infant growth**	Cross-sectional	Mothers and newborns, Australia	For the first time no significant association was found between p,p'-DDE concentrations in human milk and infant growth outcomes such as weight, length, head circumference and percentage fat mass. (N = 40)	Du, 2017 [54]

**Chemical**	**Endpoint**	**Epidemiologic design**	**Population**	**Statistically significant associations / Findings**	**First Author + Year of publication**
**p,p'-DDE**	**Cord blood transcriptome**	Cross-sectional	Newborns, Belgium	Transcription factor enrichment analysis: Glucocorticoid Receptor; Pathway Enrichment: 'insulin receptor signaling', 'acute phase response signaling', 'Interleukin(IL)-6 signaling', 'prolactin signaling'	Remy, 2016 [24]
**p,p'-DDE**	**Diabetes**	Cross-sectional	Adults, US	Differences in diabetes prevalence between quartiles of exposure	Aminov, 2016 [55]
**p,p'-DDE**	**Metabolomics**	Cross-sectional	Adults, Sweden (PIVUS)	The majority of the significant metabolites belong to lipid metabolism pathways and include fatty acids, glycerophospholipids, sphingolipids, and glycerolipids.	Salihovic, 2016 [56]
**BPA**	**Cardiometabolic impairement**	Case-control	Children and adolescents, Iran	Higher odds ratio of cardiometabolic risk factors	Mansouri, 2019 [57]
**BPA**	**Type 2 diabetes**	Case-control	Adults, India	Serum levels of BPA were significantly higher in patients with T2DM compared to control individuals and positively correlated to poor glycemic control and insulin resistance.	Soundararajan, 2019 [58]

Chemical	Endpoint	Epidemiologic design	Population	Statistically significant associations / Findings	First Author + Year of publication
**BPA**	**Birth outcomes**	Prospective (Outcome ~ BPA third trimester mother)	Mothers and newborns, Taiwan	neonatal head circumference (marginally significant)	Chang, 2019 [59]
**BPA**	**Metabolic syndrome**	Cross-sectional	Adults, South Korea (Korean National Environmental Health Survey II 2012-2014)	Metabolic syndrome (negative)	Shim, 2019 [60]
**BPA**	**Glucose**	Cross-sectional	Adults, China (e-waste recycling and reference area)	Results suggest BPA exposure might be associated with abnormal fasting blood glucose in participants living in e-waste sites	Song, 2019 [61]
**BPA**	**Type 2 diabetes**	Case-control	Adults, China	Type 2 diabetes (positive for 2nd and 3rd quartile, but not for 4th quartile)	Duan, 2018 [62]
**BPA**	**Type 2 diabetes**	Meta-analysis	16 studies; 41,320 subjects	Type 2 diabetes (positive)	Hwang, 2018 [63]
**BPA**	**Type 1 diabetes**	Case-control	Children, Turkey	No significant association with T1D; Birth Weight (negative)	Rahmani, 2018 [64]
**BPA**	**Type 2 diabetes**	Case-control	Adults, Saudi Arabia	Type 2 diabetes (positive (3rd quartile)	Li, 2018 [65]
**BPA**	**Insulin resistance**	Intervention study (not wearing and do wearing gloves 1 week)	Women, Korea (Cashiers)	Insulin (positive); Insulin resistance (positive); wearing gloves shown to be protective for exposure levels	Lee, 2018 [66]

Chemical	Endpoint	Epidemiologic design	Population	Statistically significant associations / Findings	First Author + Year of publication
**BPA**	**Glucose levels**	Prospective (Outcome ~ BPA 1st & 2nd trimester)	Pregnant women, US (Lifecodes pregnancy cohort)	No associations in the overall population. Moderately high BPA concentrations were associated with increased glucose levels among overweight/obese women	Bellavia, 2018 [67]
**BPA**	**Type 2 diabetes**	Prospective nested case-control study	Adults, China (environment, inflammation and metabolic diseases study (2008-2013))	BPA is not associated with a 5-year T2D incidence.	Shu, 2018 [68]
**BPA**	**Gestational Diabetes**	Prospective	Mothers and newborns, China	Gestational Diabetes (negative); birth weight (negative); ponderal index (negative)	Wang, 2017 [69]
**BPA**	**Glucose levels**	Prospective	Subfertile pregnant women, US	Blood glucose (positive)	Chiu, 2017 [70]
**BPA**	**Metabolic syndrome**	Cross-sectional	Adults, Prague	There was no significant relation of bisphenol A level to diabetes, hypertension, dyslipidemia, age, and BMI.	Piecha, 2017 [71]
**BPA**	**Diabetes**	Cross-sectional	Adult men, Canada	glycated hemoglobin (HbA1c, positive), diabetes melitus (positive)	Stojanoska, 2017 [72]
**BPA**	**Metabolism biomarkers**	Prospective (Outcome ~ BPA pregnancy and at	Children 8-14y, Mexico (ELEMENT)	Leptin (positive, boys)	Watkins, 2016 [73]

Chemical	Endpoint	Epidemiologic design	Population	Statistically significant associations / Findings	First Author + Year of publication
		follow-up)			

Supplementary Table 2: BPA, p,p′-DDE, PFOA, and TCS levels in the Flemish Environment And Health Studies (FLEHS) [74–81]

Chemical	CAS Number	Study Acronym + link IPCHEM metdata page	Year	Matrix	Unit of Measure	N	N Below LOD/LOQ	FREQ sex	Population	P05	P10	P25	P50	P75	P90	P95
BPA	80-5-7	FLEHS 3 Ref Adult	2014	Urine-spot	μg/L	194	7	FEMALE , N = 101 ; MALE , N = 93	Adults	0.28	0.35	0.61	1.12	1.96	3.57	5.14
BPA	80-5-7	FLEHS 3 Ref Adult	2014	Urine-spot	μg/g creatinine	194	7	FEMALE , N = 101 ; MALE , N = 93	Adults	0.40	0.63	1.02	1.57	2.58	4.17	6.44
BPA	80-5-7	FLEHS 3 Ref Adult	2014	Urine-spot	μg/L adjusted for specific gravity (SG)	194	7	FEMALE , N = 101 ; MALE , N = 93	Adults	0.53	0.82	1.44	2.10	3.50	5.74	8.14

Chemical	CAS Number	Study Acronym + link IPCHEM metdata page	Year	Matrix	Unit of Measure	N	N Below LOD/LOQ	FREQ sex	Population	P05	P10	P25	P50	P75	P90	P95
PFOA	335-67-1	FLEHS 2 Ref Nb	2008-2009	Cord blood-plasma	µg/L	220	0	FEMALE , N = 109 ; MALE , N = 111	Newbornw	0.80	0.90	1.10	1.50	2.00	2.50	2.91
PFOA	335-67-1	FLEHS 3 Ref Nb	2013-2014	Cord blood-plasma	µg/L	269	0	FEMALE , N = 130 ; MALE , N = 139	Newbornw	0.48	0.64	0.89	1.27	1.57	2.14	2.40
PFOA	335-67-1	FLEHS 2 Hotspot M	2010-2011	Blood Serum	µg/L	197	0	FEMALE , N = 83 ; MALE , N = 114	Teenagers	1.52	1.77	2.13	2.60	3.00	3.60	3.98
PFOA	335-67-1	FLEHS 2 Ref Adult	2008-2009	Blood Serum	µg/L	201	0	FEMALE , N = 107 ; MALE , N = 94	Adults	1.20	1.70	2.50	3.50	4.50	5.80	6.30
PFOA	335-67-1	FLEHS 3 Ref Adult	2014	Blood Serum	µg/L	205	0	FEMALE , N = 108 ; MALE , N = 97	Adults	1.20	1.59	2.13	2.94	3.69	4.89	6.31

Chemical	CAS Number	Study Acronym + link IPCHEM metdata page	Year	Matrix	Unit of Measure	N	N Below LOD/LOQ	FREQ sex	Population	P05	P10	P25	P50	P75	P90	P95
p,p'-DDE	72-55-9	FLEHS 1 Ref Nb	2002-2004	Cord blood-plasma	µg/L	1114	20	FEMALE , N = 532 ; MALE , N = 582	Newborns	0.05	0.07	0.13	0.22	0.38	0.62	0.91
p,p'-DDE	72-55-9	FLEHS 1 Ref Nb	2002-2004	Cord blood-plasma	µg/g lipid	1112	20	FEMALE , N = 532 ; MALE , N = 580	Newborns	0.03	0.04	0.06	0.11	0.19	0.33	0.51
p,p'-DDE	72-55-9	FLEHS 2 Ref Nb	2008-2009	Cord blood-plasma	µg/L	253	0	FEMALE , N = 125 ; MALE , N = 128	Newborns	0.06	0.07	0.09	0.15	0.24	0.38	0.52
p,p'-DDE	72-55-9	FLEHS 2 Ref Nb	2008-2009	Cord blood-plasma	µg/g lipid	250	0	FEMALE , N = 125 ; MALE , N = 125	Newborns	0.03	0.03	0.05	0.07	0.13	0.20	0.27

Chemical	CAS Number	Study Acronym + link IPCHEM metdata page	Year	Matrix	Unit of Measure	N	N Below LOD/LOQ	FREQ sex	Population	P05	P10	P25	P50	P75	P90	P95
p,p'-DDE	72-55-9	FLEHS 3 Ref Nb	2013-2014	Cord blood-plasma	µg/L	276	0	FEMALE , N = 135 ; MALE , N = 141	Newborns	0.04	0.05	0.06	0.10	0.16	0.29	0.49
p,p'-DDE	72-55-9	FLEHS 3 Ref Nb	2013-2014	Cord blood-plasma	µg/g lipid	273	0	FEMALE , N = 134 ; MALE , N = 139	Newborns	0.02	0.03	0.04	0.06	0.09	0.14	0.27
p,p'-DDE	72-55-9	FLEHS 1 Ref Ado	2003-2004	Blood Serum	µg/L	1653	1	FEMALE , N = 778 ; MALE , N = 875	Teenagers	0.15	0.19	0.27	0.41	0.72	1.50	2.30
p,p'-DDE	72-55-9	FLEHS 1 Ref Ado	2003-2004	Blood Serum	µg/g lipid	1653	1	FEMALE , N = 778 ; MALE , N = 875	Teenagers	0.03	0.04	0.06	0.09	0.16	0.33	0.52
p,p'-DDE	72-55-9	FLEHS 2 Hotspot GenkZ	2010	Blood Serum	µg/L	196	0	FEMALE , N = 107 ; MALE , N = 89	Teenagers	0.08	0.10	0.13	0.19	0.30	0.54	0.75

Chemical	CAS Number	Study Acronym + link IPCHEM metdata page	Year	Matrix	Unit of Measure	N	N Below LOD/LOQ	FREQ sex	Population	P05	P10	P25	P50	P75	P90	P95
p,p'-DDE	72-55-9	FLEHS 2 Hotspot GenkZ	2010	Blood Serum	µg/g lipid	196	0	FEMALE , N = 107 ; MALE , N = 89	Teenagers	0.02	0.02	0.03	0.04	0.07	0.11	0.18
p,p'-DDE	72-55-9	FLEHS 2 Hotspot M	2010-2011	Blood Serum	µg/L	197	0	FEMALE , N = 83 ; MALE , N = 114	Teenagers	0.08	0.10	0.13	0.19	0.32	0.54	0.75
p,p'-DDE	72-55-9	FLEHS 2 Hotspot M	2010-2011	Blood Serum	µg/g lipid	197	0	FEMALE , N = 83 ; MALE , N = 114	Teenagers	0.02	0.02	0.03	0.04	0.07	0.11	0.17
p,p'-DDE	72-55-9	FLEHS 2 Ref Ado	2008-2009	Blood Serum	µg/L	210	0	FEMALE , N = 89 ; MALE , N = 121	Teenagers	0.12	0.13	0.18	0.26	0.46	0.79	1.22
p,p'-DDE	72-55-9	FLEHS 2 Ref Ado	2008-2009	Blood Serum	µg/g lipid	208	0	FEMALE , N = 88 ; MALE , N = 120	Teenagers	0.02	0.03	0.04	0.06	0.11	0.19	0.30

Chemical	CAS Number	Study Acronym + link IPCHEM metdata page	Year	Matrix	Unit of Measure	N	N Below LOD/LOQ	FREQ sex	Population	P05	P10	P25	P50	P75	P90	P95
p,p'-DDE	72-55-9	FLEHS 3 Hotspot GKZ	2013-2014	Blood Serum	µg/L	199	0	FEMALE , N = 99 ; MALE , N = 100	Teenagers	0.07	0.08	0.12	0.19	0.35	0.93	1.40
p,p'-DDE	72-55-9	FLEHS 3 Hotspot GKZ	2013-2014	Blood Serum	µg/g lipid	198	0	FEMALE , N = 99 ; MALE , N = 99	Teenagers	0.02	0.02	0.03	0.04	0.08	0.22	0.35
p,p'-DDE	72-55-9	FLEHS 3 ref Ado	2013	Blood Serum	µg/L	205	1	FEMALE , N = 111 ; MALE , N = 94	Teenagers	0.06	0.09	0.12	0.20	0.35	0.77	1.09
p,p'-DDE	72-55-9	FLEHS 3 ref Ado	2013	Blood Serum	µg/g lipid	205	1	FEMALE , N = 111 ; MALE , N = 94	Teenagers	0.02	0.02	0.03	0.05	0.08	0.16	0.28
p,p'-DDE	72-55-9	FLEHS 1 Ref Adult	2004-2005	Blood Serum	µg/L	1577	6	FEMALE , N = 803 ; MALE , N = 774	Adults	0.54	0.82	1.60	2.92	5.60	9.95	13.00

Chemical	CAS Number	Study Acronym + link IPCHEM metdata page	Year	Matrix	Unit of Measure	N	N Below LOD/LOQ	FREQ sex	Population	P05	P10	P25	P50	P75	P90	P95
p,p'-DDE	72-55-9	FLEHS 1 Ref Adult	2004-2005	Blood Serum	µg/g lipid	1577	6	FEMALE , N = 803 ; MALE , N = 774	Adults	0.09	0.14	0.27	0.49	0.91	1.58	2.15
p,p'-DDE	72-55-9	FLEHS 3 Ref Adult	2014	Blood Serum	µg/L	206	0	FEMALE , N = 109 ; MALE , N = 97	Adults	0.26	0.34	0.72	1.37	2.44	4.20	6.00
p,p'-DDE	72-55-9	FLEHS 3 Ref Adult	2014	Blood Serum	µg/g lipid	202	0	FEMALE , N = 105 ; MALE , N = 97	Adults	0.05	0.06	0.13	0.24	0.38	0.64	1.05
TCS	3380-34-5	FLEHS 2 Ref Ado	2008-2009	Urine-morning urine	µg/L	197	0	FEMALE , N = 84 ; MALE , N = 113	Teenagers	0.22	0.30	0.56	1.29	4.89	63.52	152.49

Chemical	CAS Number	Study Acronym + link IPCHEM metdata page	Year	Matrix	Unit of Measure	N	N Below LOD/LOQ	FREQ sex	Population	P05	P10	P25	P50	P75	P90	P95
TCS	3380-34-5	FLEHS 2 Ref Ado	2008-2009	Urine-morning urine	µg/g creatinine	196	0	FEMALE , N = 84 ; MALE , N = 112	Teenagers	0.17	0.20	0.37	0.92	3.71	48.50	97.48
TCS	3380-34-5	FLEHS 2 Ref Ado	2008-2009	Urine-morning urine	µg/L adjusted for specific gravity (SG)	196	0	FEMALE , N = 84 ; MALE , N = 112	Teenagers	0.27	0.33	0.62	1.35	5.16	68.32	166.97
TCS	3380-34-5	FLEHS 3 Ref Adult	2014	Urine-spot	µg/L	194	23	FEMALE , N = 101 ; MALE , N = 93	Adults			0.18	0.39	1.30	12.08	46.12
TCS	3380-34-5	FLEHS 3 Ref Adult	2014	Urine-spot	µg/g creatinine	194	23	FEMALE , N = 101 ; MALE , N = 93	Adults			0.23	0.59	1.65	18.23	71.88

Chemical	CAS Number	Study Acronym + link IPCHEM metdata page	Year	Matrix	Unit of Measure	N	N Below LOD/LOQ	FREQ sex	Population	P05	P10	P25	P50	P75	P90	P95
TCS	3380-34-5	FLEHS 3 Ref Adult	2014	Urine-spot	µg/L adjusted for specific gravity (SG)	194	23	FEMALE , N = 101 ; MALE , N = 93	Adults			0.35	0.73	2.11	20.53	103.60

Legend: N = Number of samples; LOD = Limit of Detection; LOQ = Limit of Quantification; FREQ = Frequency table; P05, P10, P25, P50, P75, P90, P95 = 5th, 10th, 25th, 50th, 75th, 90th, 95th percentile respectively

Supplementary Table 3: Human biomonitoring data on TBT and TPP obtained from literature.

Chemical	CAS Number	Country Name	Year	Population	Matrix	Unit of Measure	Number of Samples	Median	First Author + Year of Publication
DPHP	838-85-7	China	2016-2017	e-waste dismantling area, pregnant women	Maternal Urine	μg/L	15	1.2	Bai, 2019 [82]
DPHP	838-85-7	China	2016-2017	e-waste dismantling area, pregnant women	Amnionic Fluid	μg/L	15	0.18	Bai, 2019 [82]
DPHP	838-85-7	China	2016	Children and adults	Urine, first morning	μg/L	323	0.3	Zhang, 2018 [83]
DPHP	838-85-7	US	2016	Toddlers	Urine	μg/L adjusted for SG	21	3.4	Thomas, 2017 [84]
DPHP	838-85-7	US	2016	Toddlers	Urine	μg/L adjusted for SG	20	8.2	Thomas, 2017 [84]
DPHP	838-85-7	China	2015	Pregnant women	Urine	μg/L	23	0.83	Feng, 2016 [85]
TPP	115-86-6	US	2014	Adults	Hair	ng/g	50	220	Liu, 2016 [86]
TPP	115-86-6	US	2014	Adults	Fingernail	ng/g	50	370	Liu, 2016 [86]
TPP	115-86-6	US	2014	Adults	Toenail	ng/g	50	1080	Liu, 2016 [86]
TBT	1461-22-9	US	1998	Adults	Blood	μg/L	32	5.8	Kannan, 1999 [87]
TBT	1461-22-9	Denmark	1997-2001	Newborn	Placenta	ng/g fw	129	0.01	Rantakokko, 2013 [88]

TBT	1461-22-9	Finland	1997-2001	Newborn	Placenta	ng/g fw	56	0.38	Rantakokko, 2013 [88]

Supplementary Table 4. Examples of (Q)SAR models predicting activity related to nuclear receptors involved in metabolic disruption by MDCs

Nuclear Receptor	Method	Dataset	Data type	Chemical domain	statistics	references
PPARα	multiple linear regressions (MLR) and partial least squares (PLS)	46	pEC50	phenylpropanoic acid derivatives	Training: R^2 = 0.784, Q^2= 0.774, Test: R^2= 0.841	Verma and Chouhan, 2016 [89]
	Multivariate Data Analysis	71	gene transactivation data (expressed as EC50)	carboxylic acid derivatives	Training: R^2 = 0.721, Q^2= 0.476, Blind test: Residuals Δ <1.5	Vallianatou, 2013 [90]
PPARγ	3D-QSAR	170	pEC50	PPARγ full agonists	Training: R^2 = 0.821, Q^2= 0.610, Test: R^2 = 0.552	Al Sharif, 2017 [91]
PXR	k-nearest neighbor (k-NN)	2724	competing potency to hPXR	various chemicals	Training: Ac = 0,708 Sp= 0,704 Se= 0,702	Yin, 2017 [92]
	partial logistic regression	631	human PXR binding assay	environmental chemicals	Training: Ac = 0,835 Sp= 0,85 Se= 0,82 Test: Ac= 0,696 Sp=0,839 Se=0,579	Dybdahl, 2012 [93]
CAR	3D-QSAR	35	reporter gene assay with HepG2 cells transfected	Various chemicals (including bisphenol A and	Training: R^2 = 0,99, Q^2= 0.74, Test: R^2= 0.71	Kato, 2017 [94]

			with a chimerical construct of hCAR (hCAR1þA	triphenyl phosphate)		
LXRα	multidimentional QSAR	52	LXR binding affinity	heterocyclic phenylacetic acid compounds and compounds derived from podocarpic acid	Training set: R^2=0,849 Test set: R^2= 0,744	(Spreafico, 2010)[95]
LXRβ	stepwise method combined with linear discriminant analysis	41	ABCA1 promoter activation assay	ABCA1 up-regulators	Within domain: Q = 100%, Sp= 100% Se= 100%	Chen, 2018a [96]
FXR	k-nearest neighbors based on molecular fingerprints	1224	qHTS assays for agonists and antagonists of FXR	Various chemicals	Training: Ac = 0,76 Sp= 0,80 Se= 0,73 Test: Ac=0,79 Sp=0,77 Se=0,90	Chen, 2018b [97]
	Structural fragments	103	FXR-bla transactivation assay and FXR-SRC2 coactivator assay	avermectin anthelmintics, dihydropyridine calcium channel blockers, 1,3-indandione rodenticides, and	area under the receiver operating characteristic (AUC-ROC) >0.78 (AUC≤0.5 for random data)	Hsu, 2016 [98]

	pyrethroid pesticides

Ac: predictive accuracy, Sp: specificity, Se: sensitivity

References

1. Rantakokko, P.; Main, K.M.; Wohlfart-Veje, C.; Kiviranta, H.; Airaksinen, R.; Vartiainen, T.; Skakkebæk, N.E.; Toppari, J.; Virtanen, H.E. Association of placenta organotin concentrations with growth and ponderal index in 110 newborn boys from Finland during the first 18 months of life: A cohort study. *Environ. Heal. A Glob. Access Sci. Source* **2014**, *13*.

2. Bassler, J.; Ducatman, A.; Elliott, M.; Wen, S.; Wahlang, B.; Barnett, J.; Cave, M.C. Environmental perfluoroalkyl acid exposures are associated with liver disease characterized by apoptosis and altered serum adipocytokines. *Environ. Pollut.* **2019**.

3. Rahman, M.L.; Zhang, C.; Smarr, M.M.; Lee, S.; Honda, M.; Kannan, K.; Tekola-Ayele, F.; Buck Louis, G.M. Persistent organic pollutants and gestational diabetes: A multi-center prospective cohort study of healthy US women. *Environ. Int.* **2019**, 249–258.

4. Tian, Y.-P.; Zeng, X.-W.; Bloom, M.S.; Lin, S.; Wang, S.-Q.; Yim, S.H.L.; Yang, M.; Chu, C.; Gurram, N.; Hu, L.-W.; et al. Isomers of perfluoroalkyl substances and overweight status among Chinese by sex status: Isomers of C8 Health Project in China. *Environ. Int.* **2019**, *124*, 130–138.

5. Jain, R.B.; Ducatman, A. Roles of gender and obesity in defining correlations between perfluoroalkyl substances and lipid/lipoproteins. *Sci. Total Environ.* **2019**, *653*, 74–81.

6. Liu, P.; Yang, F.; Wang, Y.; Yuan, Z. Perfluorooctanoic acid (PFOA) exposure in early life increases risk of childhood adiposity: A meta-analysis of prospective cohort studies. *Int. J. Environ. Res. Public Health* **2018**, *15*.

7. Bell, E.M.; Yeung, E.H.; Ma, W.; Kannan, K.; Sundaram, R.; Smarr, M.M.; Buck Louis, G.M. Concentrations of endocrine disrupting chemicals in newborn blood spots and infant outcomes in the upstate KIDS study. *Environ. Int.* **2018**, *121*, 232–239.

8. Mancini, F.R.; Rajaobelina, K.; Praud, D.; Dow, C.; Antignac, J.P.; Kvaskoff, M.; Severi, G.; Bonnet, F.; Boutron-Ruault, M.C.; Fagherazzi, G. Nonlinear associations between dietary exposures to perfluorooctanoic acid (PFOA) or perfluorooctane sulfonate (PFOS) and type 2 diabetes risk in women: Findings from the E3N cohort study. *Int. J. Hyg. Environ. Health* **2018**.

9. Jensen, R.C.; Glintborg, D.; Timmermann, C.A.G.; Nielsen, F.; Kyhl, H.B.; Andersen, H.R.; Grandjean, P.; Jensen, T.K.; Andersen, M. Perfluoroalkyl substances and glycemic status in pregnant Danish women: The Odense Child Cohort. *Environ. Int.* **2018**.

10. Miura, R.; Araki, A.; Miyashita, C.; Kobayashi, S.; Kobayashi, S.; Wang, S.L.; Chen, C.H.; Miyake, K.; Ishizuka, M.; Iwasaki, Y.; et al. An epigenome-wide study of cord blood DNA methylations in relation

to prenatal perfluoroalkyl substance exposure: The Hokkaido study. *Environ. Int.* **2018**.

11. Sun, Q.; Zong, G.; Valvi, D.; Nielsen, F.; Coull, B.; Grandjean, P. Plasma Concentrations of Perfluoroalkyl Substances and Risk of Type 2 Diabetes: A Prospective Investigation among U.S. Women. *Environ. Health Perspect.* **2018**, *126*, 037001.

12. Liu, G.; Dhana, K.; Furtado, J.D.; Rood, J.; Zong, G.; Liang, L.; Qi, L.; Bray, G.A.; DeJonge, L.; Coull, B.; et al. Perfluoroalkyl substances and changes in body weight and resting metabolic rate in response to weight-loss diets: A prospective study. *PLoS Med.* **2018**, *15*, e1002502.

13. Mora, A.M.; Fleisch, A.F.; Rifas-Shiman, S.L.; Woo Baidal, J.A.; Pardo, L.; Webster, T.F.; Calafat, A.M.; Ye, X.; Oken, E.; Sagiv, S.K. Early life exposure to per- and polyfluoroalkyl substances and mid-childhood lipid and alanine aminotransferase levels. *Environ. Int.* **2018**, *111*, 1–13.

14. Matilla-Santander, N.; Valvi, D.; Lopez-Espinosa, M.-J.; Manzano-Salgado, C.B.; Ballester, F.; Ibarluzea, J.; Santa-Marina, L.; Schettgen, T.; Guxens, M.; Sunyer, J.; et al. Exposure to Perfluoroalkyl Substances and Metabolic Outcomes in Pregnant Women: Evidence from the Spanish INMA Birth Cohorts. *Environ. Health Perspect.* **2017**, *125*, 117004.

15. Yang, Q.; Guo, X.; Sun, P.; Chen, Y.; Zhang, W.; Gao, A. Association of serum levels of perfluoroalkyl substances (PFASs) with the metabolic syndrome (MetS) in Chinese male adults: A cross-sectional study. *Sci. Total Environ.* **2018**, *621*, 1542–1549.

16. Khalil, N.; Ebert, J.R.; Honda, M.; Lee, M.; Nahhas, R.W.; Koskela, A.; Hangartner, T.; Kannan, K. Perfluoroalkyl substances, bone density, and cardio-metabolic risk factors in obese 8–12 year old children: A pilot study. *Environ. Res.* **2018**.

17. Cardenas, A.; Gold, D.R.; Hauser, R.; Kleinman, K.P.; Hivert, M.-F.; Calafat, A.M.; Ye, X.; Webster, T.F.; Horton, E.S.; Oken, E. Plasma Concentrations of Per- and Polyfluoroalkyl Substances at Baseline and Associations with Glycemic Indicators and Diabetes Incidence among High-Risk Adults in the Diabetes Prevention Program Trial. *Environ. Health Perspect.* **2017**, *125*, 107001.

18. Manzano-Salgado, C.B.; Casas, M.; Lopez-Espinosa, M.-J.; Ballester, F.; Iñiguez, C.; Martinez, D.; Romaguera, D.; Fernández-Barrés, S.; Santa-Marina, L.; Basterretxea, M.; et al. Prenatal Exposure to Perfluoroalkyl Substances and Cardiometabolic Risk in Children from the Spanish INMA Birth Cohort Study. *Environ. Health Perspect.* **2017**, *125*, 097018.

19. Liu, H.S.; Wen, L.L.; Chu, P.L.; Lin, C.Y. Association among total serum isomers of perfluorinated chemicals, glucose homeostasis, lipid profiles, serum protein and metabolic syndrome in adults: NHANES, 2013–2014. *Environ. Pollut.* **2018**.

20. Starling, A.P.; Adgate, J.L.; Hamman, R.F.; Kechris, K.; Calafat, A.M.; Ye, X.; Dabelea, D. Perfluoroalkyl Substances during Pregnancy and Offspring Weight and Adiposity at Birth: Examining Mediation by Maternal Fasting Glucose in the Healthy Start Study. *Environ. Health Perspect.* **2017**, *125*, 067016.

21. Kingsley, S.L.; Kelsey, K.T.; Butler, R.; Chen, A.; Eliot, M.N.; Romano, M.E.; Houseman, A.; Koestler, D.C.; Lanphear, B.P.; Yolton, K.; et al. Maternal serum PFOA concentration and DNA methylation in cord blood: A pilot study. *Environ. Res.* **2017**, *158*, 174–178.

22. Hartman, T.J.; Calafat, A.M.; Holmes, A.K.; Marcus, M.; Northstone, K.; Flanders, W.D.; Kato, K.; Taylor, E. V Prenatal Exposure to Perfluoroalkyl Substances and Body Fatness in Girls. *Child. Obes.* **2017**, *13*, 222–230.

23. Lauritzen, H.B.; Larose, T.L.; Øien, T.; Sandanger, T.M.; Odland, J.Ø.; van de Bor, M.; Jacobsen, G.W. Maternal serum levels of perfluoroalkyl substances and organochlorines and indices of fetal growth: a Scandinavian case-cohort study. *Pediatr. Res.* **2017**, *81*, 33–42.

24. Remy, S.; Govarts, E.; Wens, B.; De Boever, P.; Den Hond, E.; Croes, K.; Sioen, I.; Baeyens, W.; van Larebeke, N.; Koppe, J.; et al. Metabolic targets of endocrine disrupting chemicals assessed by cord blood transcriptome profiling. *Reprod. Toxicol.* **2016**.

25. Fleisch, A.F.; Rifas-Shiman, S.L.; Mora, A.M.; Calafat, A.M.; Ye, X.; Luttmann-Gibson, H.; Gillman, M.W.; Oken, E.; Sagiv, S.K. Early-Life Exposure to Perfluoroalkyl Substances and Childhood Metabolic Function. *Environ. Health Perspect.* **2017**, *125*, 481–487.

26. Domazet, S.L.; GrØntved, A.; Timmermann, A.G.; Nielsen, F.; Jensen, T.K. Longitudinal associations of exposure to perfluoroalkylated substances in childhood and adolescence and indicators of adiposity and glucose metabolism 6 and 12 years later: The European youth heart study. *Diabetes Care* **2016**, *39*, 1745–1751.

27. Mora, A.M.; Oken, E.; Rifas-Shiman, S.L.; Webster, T.F.; Gillman, M.W.; Calafat, A.M.; Ye, X.; Sagiv, S.K. Prenatal Exposure to Perfluoroalkyl Substances and Adiposity in Early and Mid-Childhood. *Environ. Health Perspect.* **2017**, *125*, 467–473.

28. Conway, B.; Innes, K.E.; Long, D. Perfluoroalkyl substances and beta cell deficient diabetes. *J. Diabetes Complications* **2016**, *30*, 993–8.

29. Su, T.C.; Kuo, C.C.; Hwang, J.J.; Lien, G.W.; Chen, M.F.; Chen, P.C. Serum perfluorinated chemicals, glucose homeostasis and the risk of diabetes in working-aged Taiwanese adults. *Environ. Int.* **2016**.

30. Braun, J.M.; Chen, A.; Romano, M.E.; Calafat, A.M.; Webster, G.M.; Yolton, K.; Lanphear, B.P. Prenatal perfluoroalkyl substance exposure and child adiposity at 8 years of age: The HOME study. *Obesity (Silver Spring).* **2016**, *24*, 231–7.

31. Liu, H.; Li, J.; Xia, W.; Zhang, B.; Peng, Y.; Li, Y.; Zhou, Y.; Fang, J.; Zhao, H.; Jiang, Y.; et al. Blood pressure changes during pregnancy in relation to urinary paraben, triclosan and benzophenone concentrations: A repeated measures study. *Environ. Int.* **2019**, *122*, 185–192.

32. Wu, C.; Li, J.; Xia, W.; Li, Y.; Zhang, B.; Zhou, A.; Hu, J.; Li, C.; Zhao, H.; Jiang, M.; et al. The association of repeated measurements of prenatal exposure to triclosan with fetal and early-childhood growth.

Environ. Int. **2018**, *120*, 54–62.

33. Kalloo, G.; Calafat, A.M.; Chen, A.; Yolton, K.; Lanphear, B.P.; Braun, J.M. Early life Triclosan exposure and child adiposity at 8 Years of age: A prospective cohort study. *Environ. Heal. A Glob. Access Sci. Source* **2018**, *17*.

34. Ouyang, F.; Tang, N.; Zhang, H.-J.; Wang, X.; Zhao, S.; Wang, W.; Zhang, J.; Cheng, W. Maternal urinary triclosan level, gestational diabetes mellitus and birth weight in Chinese women. *Sci. Total Environ.* **2018**, *626*, 451–457.

35. Ferguson, K.K.; Meeker, J.D.; Cantonwine, D.E.; Mukherjee, B.; Pace, G.G.; Weller, D.; McElrath, T.F. Environmental phenol associations with ultrasound and delivery measures of fetal growth. *Environ. Int.* **2018**, *112*, 243–250.

36. Shapiro, G.D.; Arbuckle, T.E.; Ashley-Martin, J.; Fraser, W.D.; Fisher, M.; Bouchard, M.F.; Monnier, P.; Morisset, A.-S.; Ettinger, A.S.; Dodds, L. Associations between maternal triclosan concentrations in early pregnancy and gestational diabetes mellitus, impaired glucose tolerance, gestational weight gain and fetal markers of metabolic function. *Environ. Res.* **2018**, *161*, 554–561.

37. Huo, W.; Xia, W.; Wu, C.; Zhu, Y.; Zhang, B.; Wan, Y.; Zhou, A.; Qian, Z.; Chen, Z.; Jiang, Y.; et al. Urinary level of triclosan in a population of Chinese pregnant women and its association with birth outcomes. *Environ. Pollut.* **2018**, *233*, 872–879.

38. Deierlein, A.L.; Wolff, M.S.; Pajak, A.; Pinney, S.M.; Windham, G.C.; Galvez, M.P.; Rybak, M.; Calafat, A.M.; Kushi, L.H.; Biro, F.M.; et al. Phenol Concentrations During Childhood and Subsequent Measures of Adiposity Among Young Girls. *Am. J. Epidemiol.* **2017**, *186*, 581–592.

39. Geer, L.A.; Pycke, B.F.G.; Waxenbaum, J.; Sherer, D.M.; Abulafia, O.; Halden, R.U. Association of birth outcomes with fetal exposure to parabens, triclosan and triclocarban in an immigrant population in Brooklyn, New York. *J. Hazard. Mater.* **2017**, *323*, 177–183.

40. Buckley, J.P.; Herring, A.H.; Wolff, M.S.; Calafat, A.M.; Engel, S.M. Prenatal exposure to environmental phenols and childhood fat mass in the Mount Sinai Children's Environmental Health Study. *Environ. Int.* **2016**, *91*, 350–6.

41. Lassen, T.H.; Frederiksen, H.; Kyhl, H.B.; Swan, S.H.; Main, K.M.; Andersson, A.-M.; Lind, D.V.; Husby, S.; Wohlfahrt-Veje, C.; Skakkebæk, N.E.; et al. Prenatal Triclosan Exposure and Anthropometric Measures Including Anogenital Distance in Danish Infants. *Environ. Health Perspect.* **2016**, *124*, 1261–8.

42. Donat-Vargas, C.; Akesson, A.; Tornevi, A.; Wennberg, M.; Sommar, J.; Kiviranta, H.; Rantakokko, P.; Bergdahl, I.A. Persistent organochlorine pollutants in plasma, blood pressure, and hypertension in a longitudinal study. *Hypertension* **2018**, *71*, 1258–1268.

43. Zong, G.; Valvi, D.; Coull, B.; Göen, T.; Hu, F.B.; Nielsen, F.; Grandjean, P.; Sun, Q. Persistent organic pollutants and risk of type 2 diabetes: A prospective investigation among middle-aged women in

Nurses' Health Study II. *Environ. Int.* **2018**, *114*, 334–342.

44. Coker, E.; Chevrier, J.; Rauch, S.; Bradman, A.; Obida, M.; Crause, M.; Bornman, R.; Eskenazi, B. Association between prenatal exposure to multiple insecticides and child body weight and body composition in the VHEMBE South African birth cohort. *Environ. Int.* **2018**, *113*, 122–132.

45. La Merrill, M.A.; Lind, P.M.; Salihovic, S.; van Bavel, B.; Lind, L. The association between p,p′-DDE levels and left ventricular mass is mainly mediated by obesity. *Environ. Res.* **2018**, *160*, 541–546.

46. Cano-Sancho, G.; Salmon, A.G.; La Merrill, M.A. Association between Exposure to p,p′-DDT and Its Metabolite p,p′-DDE with Obesity: Integrated Systematic Review and Meta-Analysis. *Environ. Health Perspect.* **2017**, *125*, 096002.

47. Warner, M.; Ye, M.; Harley, K.; Kogut, K.; Bradman, A.; Eskenazi, B. Prenatal DDT exposure and child adiposity at age 12: The CHAMACOS study. *Environ. Res.* **2017**, *159*, 606–612.

48. Rosenbaum, P.F.; Weinstock, R.S.; Silverstone, A.E.; Sjödin, A.; Pavuk, M. Metabolic syndrome is associated with exposure to organochlorine pesticides in Anniston, AL, United States. *Environ. Int.* **2017**, *108*, 11–21.

49. Valvi, D.; Oulhote, Y.; Weihe, P.; Dalgård, C.; Bjerve, K.S.; Steuerwald, U.; Grandjean, P. Gestational diabetes and offspring birth size at elevated environmental pollutant exposures. *Environ. Int.* **2017**, *107*, 205–215.

50. Singh, K.; Chan, H.M. Persistent organic pollutants and diabetes among Inuit in the Canadian Arctic. *Environ. Int.* **2017**, *101*, 183–189.

51. Artacho-Cordón, F.; León, J.; Sáenz, J.M.; Fernández, M.F.; Martin-Olmedo, P.; Olea, N.; Arrebola, J.P. Contribution of Persistent Organic Pollutant Exposure to the Adipose Tissue Oxidative Microenvironment in an Adult Cohort: A Multipollutant Approach. *Environ. Sci. Technol.* **2016**, *50*, 13529–13538.

52. Xu, C.; Yin, S.; Tang, M.; Liu, K.; Yang, F.; Liu, W. Environmental exposure to DDT and its metabolites in cord serum: Distribution, enantiomeric patterns, and effects on infant birth outcomes. *Sci. Total Environ.* **2017**, *580*, 491–498.

53. Carrizo, D.; Chevallier, O.P.; Woodside, J. V; Brennan, S.F.; Cantwell, M.M.; Cuskelly, G.; Elliott, C.T. Untargeted metabolomic analysis of human serum samples associated with exposure levels of Persistent organic pollutants indicate important perturbations in Sphingolipids and Glycerophospholipids levels. *Chemosphere* **2017**, *168*, 731–738.

54. Du, J.; Gridneva, Z.; Gay, M.C.L.; Trengove, R.D.; Hartmann, P.E.; Geddes, D.T. Pesticides in human milk of Western Australian women and their influence on infant growth outcomes: A cross-sectional study. *Chemosphere* **2017**, *167*, 247–254.

55. Aminov, Z.; Haase, R.; Rej, R.; Schymura, M.J.; Santiago-Rivera, A.; Morse, G.; DeCaprio, A.; Carpenter,

D.O.; Akwesasne Task Force on the Environment Diabetes Prevalence in Relation to Serum Concentrations of Polychlorinated Biphenyl (PCB) Congener Groups and Three Chlorinated Pesticides in a Native American Population. *Environ. Health Perspect.* **2016**, *124*, 1376–83.

56. Salihovic, S.; Ganna, A.; Fall, T.; Broeckling, C.D.; Prenni, J.E.; van Bavel, B.; Lind, P.M.; Ingelsson, E.; Lind, L. The metabolic fingerprint of p,p′-DDE and HCB exposure in humans. *Environ. Int.* **2016**, *88*, 60–66.

57. Mansouri, V.; Ebrahimpour, K.; Poursafa, P.; Riahi, R.; Shoshtari-Yeganeh, B.; Hystad, P.; Kelishadi, R. Exposure to phthalates and bisphenol A is associated with higher risk of cardiometabolic impairment in normal weight children. *Environ. Sci. Pollut. Res. Int.* **2019**, *26*, 18604–18614.

58. Soundararajan, A.; Prabu, P.; Mohan, V.; Gibert, Y.; Balasubramanyam, M. Novel insights of elevated systemic levels of bisphenol-A (BPA) linked to poor glycemic control, accelerated cellular senescence and insulin resistance in patients with type 2 diabetes. *Mol. Cell. Biochem.* **2019**, *458*, 171–183.

59. Chang, C.-H.; Huang, Y.-F.; Wang, P.-W.; Lai, C.-H.; Huang, L.-W.; Chen, H.-C.; Lin, M.-H.; Yang, W.; Mao, I.-F.; Chen, M.-L. Associations between prenatal exposure to bisphenol a and neonatal outcomes in a Taiwanese cohort study: Mediated through oxidative stress? *Chemosphere* **2019**, *226*, 290–297.

60. Shim, Y.H.; Ock, J.W.; Kim, Y.-J.; Kim, Y.; Kim, S.Y.; Kang, D. Association between Heavy Metals, Bisphenol A, Volatile Organic Compounds and Phthalates and Metabolic Syndrome. *Int. J. Environ. Res. Public Health* **2019**, *16*.

61. Song, S.; Duan, Y.; Zhang, T.; Zhang, B.; Zhao, Z.; Bai, X.; Xie, L.; He, Y.; Ouyang, J.-P.; Huang, X.; et al. Serum concentrations of bisphenol A and its alternatives in elderly population living around e-waste recycling facilities in China: Associations with fasting blood glucose. *Ecotoxicol. Environ. Saf.* **2019**, *169*, 822–828.

62. Duan, Y.; Yao, Y.; Wang, B.; Han, L.; Wang, L.; Sun, H.; Chen, L. Association of urinary concentrations of bisphenols with type 2 diabetes mellitus: A case-control study. *Environ. Pollut.* **2018**, 1719–1726.

63. Hwang, S.; Lim, J.-E.; Choi, Y.; Jee, S.H. Bisphenol A exposure and type 2 diabetes mellitus risk: a meta-analysis. *BMC Endocr. Disord.* **2018**, *18*, 81.

64. Rahmani, S.; Pour Khalili, N.; Khan, F.; Hassani, S.; Ghafour-Boroujerdi, E.; Abdollahi, M. Bisphenol A: What lies beneath its induced diabetes and the epigenetic modulation? *Life Sci.* **2018**, *214*, 136–144.

65. Li, A.J.; Xue, J.; Lin, S.; Al-Malki, A.L.; Al-Ghamdi, M.A.; Kumosani, T.A.; Kannan, K. Urinary concentrations of environmental phenols and their association with type 2 diabetes in a population in Jeddah, Saudi Arabia. *Environ. Res.* **2018**, *166*, 544–552.

66. Lee, I.; Kim, S.; Kim, K.-T.; Kim, S.; Park, S.; Lee, H.; Jeong, Y.; Lim, J.-E.; Moon, H.-B.; Choi, K. Bisphenol A exposure through receipt handling and its association with insulin resistance among female cashiers. *Environ. Int.* **2018**, *117*, 268–275.

67. Bellavia, A.; Cantonwine, D.E.; Meeker, J.D.; Hauser, R.; Seely, E.W.; McElrath, T.F.; James-Todd, T. Pregnancy urinary bisphenol-A concentrations and glucose levels across BMI categories. *Environ. Int.* **2018**, *113*, 35–41.

68. Shu, X.; Tang, S.; Peng, C.; Gao, R.; Yang, S.; Luo, T.; Cheng, Q.; Wang, Y.; Wang, Z.; Zhen, Q.; et al. Bisphenol A is not associated with a 5-year incidence of type 2 diabetes: a prospective nested case-control study. *Acta Diabetol.* **2018**, *55*, 369–375.

69. Wang, X.; Wang, X.; Chen, Q.; Luo, Z.-C.; Zhao, S.; Wang, W.; Zhang, H.-J.; Zhang, J.; Ouyang, F. Urinary Bisphenol A Concentration and Gestational Diabetes Mellitus in Chinese Women. *Epidemiology* **2017**, *28 Suppl 1*, S41–S47.

70. Chiu, Y.H.; Mínguez-Alarcón, L.; Ford, J.B.; Keller, M.; Seely, E.W.; Messerlian, C.; Petrozza, J.; Williams, P.L.; Ye, X.; Calafat, A.M.; et al. Trimester-specific urinary bisphenol a concentrations and blood glucose levels among pregnant women from a fertility clinic. *J. Clin. Endocrinol. Metab.* **2017**, *102*, 1350–1357.

71. Piecha, R.; Svačina, Š.; Malý, M.; Vrbík, K.; Lacinová, Z.; Haluzík, M.; Pavloušková, J.; Vavrouš, A.; Matějková, D.; Müllerová, D.; et al. Urine Levels of Phthalate Metabolites and Bisphenol A in Relation to Main Metabolic Syndrome Components: Dyslipidemia, Hypertension and Type 2 Diabetes. A Pilot Study. *Cent. Eur. J. Public Health* **2016**, *24*, 297–301.

72. Stojanoska, M.M.; Milosevic, N.; Milic, N.; Abenavoli, L. The influence of phthalates and bisphenol A on the obesity development and glucose metabolism disorders. *Endocrine* **2017**, *55*, 666–681.

73. Watkins, D.J.; Wellenius, G.A.; Butler, R.A.; Bartell, S.M.; Fletcher, T.; Kelsey, K.T. Associations between serum perfluoroalkyl acids and LINE-1 DNA methylation. *Environ. Int.* **2014**, *63*, 71–6.

74. De Craemer, S.; Croes, K.; van Larebeke, N.; De Henauw, S.; Schoeters, G.; Govarts, E.; Loots, I.; Nawrot, T.; Nelen, V.; Den Hond, E.; et al. Metals, hormones and sexual maturation in Flemish adolescents in three cross-sectional studies (2002–2015). *Environ. Int.* **2017**, *102*, 190–199.

75. Schoeters, G.; Govarts, E.; Bruckers, L.; Den Hond, E.; Nelen, V.; De Henauw, S.; Sioen, I.; Nawrot, T.S.; Plusquin, M.; Vriens, A.; et al. Three cycles of human biomonitoring in Flanders − Time trends observed in the Flemish Environment and Health Study. *Int. J. Hyg. Environ. Health* **2017**, *220*, 36–45.

76. Baeyens, W.; Vrijens, J.; Gao, Y.; Croes, K.; Schoeters, G.; Den Hond, E.; Sioen, I.; Bruckers, L.; Nawrot, T.; Nelen, V.; et al. Trace metals in blood and urine of newborn/mother pairs, adolescents and adults of the Flemish population (2007-2011). *Int. J. Hyg. Environ. Health* **2014**, *217*, 878–890.

77. Croes, K.; Den Hond, E.; Bruckers, L.; Loots, I.; Morrens, B.; Nelen, V.; Colles, A.; Schoeters, G.; Sioen, I.; Covaci, A.; et al. Monitoring chlorinated persistent organic pollutants in adolescents in Flanders (Belgium): Concentrations, trends and dose-effect relationships (FLEHS II). *Environ. Int.* **2014**, *71*, 20–28.

78. Den Hond, E.; Paulussen, M.; Geens, T.; Bruckers, L.; Baeyens, W.; David, F.; Dumont, E.; Loots, I.;

Morrens, B.; de Bellevaux, B.N.; et al. Biomarkers of human exposure to personal care products: Results from the Flemish Environment and Health Study (FLEHS 2007-2011). *Sci. Total Environ.* **2013**, *463–464*, 102–110.

79. Vrijens, J.; Leermakers, M.; Stalpaert, M.; Schoeters, G.; Den Hond, E.; Bruckers, L.; Colles, A.; Nelen, V.; Van Den Mieroop, E.; Van Larebeke, N.; et al. Trace metal concentrations measured in blood and urine of adolescents in Flanders, Belgium: Reference population and case studies Genk-Zuid and Menen. *Int. J. Hyg. Environ. Health* **2014**, *217*, 515–527.

80. Koppen, G.; Covaci, A.; Van Cleuvenbergen, R.; Schepens, P.; Winneke, G.; Nelen, V.; van Larebeke, N.; Vlietinck, R.; Schoeters, G.; Cleuvenbergen, R. Van Persistent organochlorine pollutants in human serum of 50 – 65 years old women in the Flanders Environmental and Health Study (FLEHS). Part 1 : concentrations and regional differences. *Chemosphere* **2002**, *48*, 811–825.

81. Koppen, G.; Den Hond, E.; Nelen, V.; Van De Mieroop, E.; Bruckers, L.; Bilau, M.; Keune, H.; Van Larebeke, N.; Covaci, A.; Van De Weghe, H.; et al. Organochlorine and heavy metals in newborns: Results from the Flemish Environment and Health Survey (FLEHS 2002-2006). *Environ. Int.* **2009**, *35*, 1015–1022.

82. Bai, X.-Y.; Lu, S.-Y.; Xie, L.; Zhang, B.; Song, S.-M.; He, Y.; Ouyang, J.-P.; Zhang, T. A pilot study of metabolites of organophosphorus flame retardants in paired maternal urine and amniotic fluid samples: potential exposure risks of tributyl phosphate to pregnant women. *Environ. Sci. Process. Impacts* **2019**, *21*, 124–132.

83. Zhang, T.; Bai, X.-Y.; Lu, S.-Y.; Zhang, B.; Xie, L.; Zheng, H.-C.; Jiang, Y.-C.; Zhou, M.-Z.; Zhou, Z.-Q.; Song, S.-M.; et al. Urinary metabolites of organophosphate flame retardants in China: Health risk from tris(2-chloroethyl) phosphate (TCEP) exposure. *Environ. Int.* **2018**, *121*, 1363–1371.

84. Thomas, M.B.; Stapleton, H.M.; Dills, R.L.; Violette, H.D.; Christakis, D.A.; Sathyanarayana, S. Demographic and dietary risk factors in relation to urinary metabolites of organophosphate flame retardants in toddlers. *Chemosphere* **2017**.

85. Feng, L.; Ouyang, F.; Liu, L.; Wang, X.; Wang, X.; Li, Y.-J.; Murtha, A.; Shen, H.; Zhang, J.; Zhang, J.J. Levels of Urinary Metabolites of Organophosphate Flame Retardants, TDCIPP, and TPHP, in Pregnant Women in Shanghai. *J. Environ. Public Health* **2016**, *2016*, 9416054.

86. Liu, L.-Y.; He, K.; Hites, R.A.; Salamova, A. Hair and Nails as Noninvasive Biomarkers of Human Exposure to Brominated and Organophosphate Flame Retardants. *Environ. Sci. Technol.* **2016**, *50*, 3065–73.

87. Kannan, K.; Senthilkumar, K.; Giesy, J.P. Occurrence of butyltin compounds in human blood. *Environ. Sci. Technol.* **1999**, *33*, 1776–1779.

88. Rantakokko, P.; Kumar, E.; Braber, J.; Huang, T.; Kiviranta, H.; Cequier, E.; Thomsen, C. Concentrations of brominated and phosphorous flame retardants in Finnish house dust and insights into children's

exposure. *Chemosphere* **2019**, 99–107.

89. Verma, N.; Chouhan, U. Chemometric Modelling of PPAR-α and PPAR-γ Dual Agonists for the Treatment of Type-2 Diabetes. *Curr. Sci.* **2016**, *111*, 356.

90. Vallianatou, T.; Lambrinidis, G.; Giaginis, C.; Mikros, E.; Tsantili-Kakoulidou, A. Analysis of PPAR-α/γ Activity by Combining 2-D QSAR and Molecular Simulation. *Mol. Inform.* **2013**, *32*, 431–45.

91. Al Sharif, M.; Tsakovska, I.; Pajeva, I.; Alov, P.; Fioravanzo, E.; Bassan, A.; Kovarich, S.; Yang, C.; Mostrag-Szlichtyng, A.; Vitcheva, V.; et al. The application of molecular modelling in the safety assessment of chemicals: A case study on ligand-dependent PPARγ dysregulation. *Toxicology* **2017**, *392*, 140–154.

92. Yin, C.; Yang, X.; Wei, M.; Liu, H. Predictive models for identifying the binding activity of structurally diverse chemicals to human pregnane X receptor. *Environ. Sci. Pollut. Res. Int.* **2017**, *24*, 20063–20071.

93. Dybdahl, M.; Nikolov, N.G.; Wedebye, E.B.; Jónsdóttir, S.Ó.; Niemelä, J.R. QSAR model for human pregnane X receptor (PXR) binding: screening of environmental chemicals and correlations with genotoxicity, endocrine disruption and teratogenicity. *Toxicol. Appl. Pharmacol.* **2012**, *262*, 301–9.

94. Kato, H.; Yamaotsu, N.; Iwazaki, N.; Okamura, S.; Kume, T.; Hirono, S. Precise prediction of activators for the human constitutive androstane receptor using structure-based three-dimensional quantitative structure–activity relationship methods. *Drug Metab. Pharmacokinet.* **2017**, *32*, 179–188.

95. Spreafico, M.; Smiesko, M.; Peristera, O.; Rossato, G.; Vedani, A. Probing Small-Molecule Binding to the Liver-X Receptor: A Mixed-Model QSAR Study. *Mol. Inform.* **2010**, *29*, 27–36.

96. Chen, M.; Yang, F.; Kang, J.; Gan, H.; Yang, X.; Lai, X.; Gao, Y. Identfication of Potent LXRβ-Selective Agonists without LXRα Activation by In Silico Approaches. *Molecules* **2018**, *23*.

97. Chen, Y.; Yang, H.; Wu, Z.; Liu, G.; Tang, Y.; Li, W. Prediction of Farnesoid X Receptor Disruptors with Machine Learning Methods. *Chem. Res. Toxicol.* **2018**, *31*, 1128–1137.

98. Hsu, C.-W.; Hsieh, J.-H.; Huang, R.; Pijnenburg, D.; Khuc, T.; Hamm, J.; Zhao, J.; Lynch, C.; van Beuningen, R.; Chang, X.; et al. Differential modulation of FXR activity by chlorophacinone and ivermectin analogs. *Toxicol. Appl. Pharmacol.* **2016**, *313*, 138–148.

© 2020 by the authors. Submitted for possible open access publication under the terms and conditions of the Creative Commons Attribution (CC BY) license (http://creativecommons.org/licenses/by/4.0/).
